# CRISPR/Cas9-mediated editing of the *GhJAZ2* gene improves fiber length and lint percentage in *Gossypium hirsutum* L

**DOI:** 10.1080/21645698.2026.2660546

**Published:** 2026-04-19

**Authors:** Muhammad Sulyman Saleem, Sultan Habibullah Khan, Iqrar Ahmad Rana, Aftab Ahmad

**Affiliations:** a Centre of Agricultural Biochemistry and Biotechnology (CABB), University of Agriculture Faisalabad, Faisalabad, Pakistan; b Center for Advanced Studies in Agriculture and Food Security (CAS-AFS), University of Agriculture Faisalabad, Faisalabad, Pakistan; c Natural and Medical Sciences Research Center (NMSRC), University of Nizwa, Nizwa, Oman; d Department of Biochemistry, University of Agriculture Faisalabad, Faisalabad, Pakistan

**Keywords:** Cotton, CRISPR/Cas9, fiber traits, gene editing, GhJAZ2, in planta transformation

## Abstract

Cotton is regarded as a strategic agricultural commodity owing to its renewable and naturally derived fiber. With the escalating global demand for high-quality fiber, genetic improvement of fiber traits is a critical focus for sustaining and advancing the textile industry standards. The cotton *GhJAZ2* gene encodes the Jasmonate ZIM-domain 2 protein, a known repressor in the jasmonic acid signaling pathway and negatively regulates fiber initiation. In this study, we designed a gRNA that simultaneously targets *GhJAZ2* homologs and assembled it into the CRISPR vector (pHSE401). Subsequently, the construct (pHSE401-gRNA) was transformed into cotton (*Gossypium hirsutum* L.) using an Agrobacterium-mediated *in planta* transformation strategy, targeting the shoot apical meristem as the primary site of transformation. Sanger sequencing analysis revealed consistent single-base pair indels at the targeted site across both A and D sub-genomes, with edited T_1_ progenies showing both inherited and newly introduced indels at the targeted loci. Fiber analysis of edited lines compared to the control revealed a significant (*p < .05*) enhancement in lint percentage (≤13.74%) and fiber length (≤16.91%). This study demonstrated the effective application of CRISPR/Cas9 for targeted trait improvement in cotton, offering *GhJAZ2*-edited lines that can be advanced to develop transgene-free cultivars with improved fiber traits.

## 1. Introduction

Cotton, a member of the genus Gossypium in the family Malvaceae, is the world’s leading renewable, natural fiber, supplying approximately 40% of the annual fiber demand in the textile industry.^1^ Staple length is a key factor in defining the quality of cotton fiber in the global textile industry. Cotton fiber morphogenesis contains various differentiation phases, such as initiation, elongation, transition, secondary wall synthesis, and maturation, which commonly take at least 50 days.^2^ The seed’s surface has a single epidermis from which each long cotton lint fiber grows, which thickens its cell wall to become a very long and strong dead fiber in cotton.^3–5^ The number of fibers begun on the seed’s outer surface is the primary determinant of fiber yield, and these differentiation processes directly influence the qualitative traits of cotton fiber.^6^

Jasmonic acid (JA) is essential for regulating plant growth and defense mechanisms, anthocyanin accumulation, including trichome and fiber development.^7^ A high concentration of JA significantly represses fiber initiation, whereas an optimal concentration promotes fiber elongation in cultured ovules.^8^ Modulation of JA and the molecular mechanism of fiber initiation may be potential factors for improving length and quality. JA regulates fiber initiation by targeting specific transcription factors that control the expression of associated genes within the JA signaling pathway. The cotton *GhJAZ2* gene-encoded JAZ2 (Jasmonate ZIM-domain 2) protein acts as a repressor in the JA signaling pathway and contributes to lint and fuzz fiber initiation.^9^

In the absence of JA, GhJAZ2 inhibits the transactivation activity of GhMYB25-like, GhGL1 (bHLH transcription factor), GhMYC2, and GhWD40, which are the main constituents of the WD-repeat/bHLH/MYB transcriptional complex.^10^ But in the presence of JA, the SCFCOI1 complex recruits GhJAZ2 for ubiquitination and degradation via the 26S proteasome, leading to activation of these transcription factors and fiber initiation.^11^ In cotton, the *GhJAZ2* gene is highly expressed in the ovules during the fiber initiation stage (0 days post-anthesis, DPA).^10^ Its overexpression inhibits fiber initiation and reduces fiber length by repressing the transcription factors, including GhMYB25-like, GhGL1, GhMYC2, GhWD40, and GhJI within the JA signaling pathway.^10^

The CRISPR/Cas9 system has been successfully employed as a versatile and efficient tool for gene editing in cotton.^12^ The CRISPR/Cas9 system contains a guide RNA (gRNA) that recognizes the target sequence and a Cas9 nuclease that cleaves the target DNA sequence at a specific location in the targeted genome to induce a double-strand break (DSB), which can be repaired by homologous recombination (HR), like homologous directed repair (HDR) or non-homologous end joining (NHEJ), resulting in random insertion or deletion of nucleotides in the case of NHEJ.^13^

CRISPR/Cas9 reagents transformation into the cotton plant is critical, which can be mediated by Agrobacterium^14–18^ or plant viruses.^19–21^ In the case of plant viruses, many replicating viral vectors cannot accommodate the Cas9 gene due to its considerably large size. They also cannot target the meristematic cells to induce mutations and give heritable edits from seeds. Hence, the agrobacterium-mediated transformation strategy is most commonly and broadly applicable for cotton plants due to its simplicity, cost-effectiveness, stable, single-copy gene integration, and higher editing efficiency.

The Agrobacterium-mediated transformation strategy has been predominantly employed with somatic embryogenesis and shoot organogenesis. However, genotype-dependency and in vitro regeneration require tedious tissue culture procedures and a long time to regenerate into a whole cotton plant.^22^ Chen et al.^23^ and Ge et al.^24^ employed a genotype-independent but tissue culture-dependent shoot apical meristem cell-mediated transformation approach to transform recalcitrant cotton genotypes and edit them with heritable targeted editing in multiple genes via the CRISPR/Cas9 system. However, some research groups have also adopted such shoot apical meristem-targeted *in planta* transformation strategy to transform cotton plants,^25^^–^^28^ which is a genotype and tissue culture-independent, cost-effective, and time-saving methodology by eliminating obstacles associated with somatic embryogenesis and shoot organogenesis.

Thus, editing cotton fiber-related genes using the CRISPR/Cas9 tool is necessary to meet the increasing demand for cotton with superior fiber quality as a natural textile fiber. In the current study, a CRISPR/Cas9-based vector (pHSE401-gRNA) was constructed to edit the cotton *GhJAZ2* gene, transformed the cotton plants (*G. hirsutum* L.) using a genotype and tissue culture-independent *in planta* transformation strategy using the shoot apical meristem, and cotton transformants were screened for the edited *GhJAZ2* gene and improved fiber traits.

## 2. Materials and Methods

### 2.1. Guide-RNA Designing and Off-Target Analysis

The nucleotide sequences of the target *GhJAZ2* gene, including exons and introns from both the A_t_ sub-genome (*GhJAZ2-A*; Gh_A06G0705) and the D_t_ sub-genome (*GhJAZ2-D*; Gh_D06G0810), were retrieved from the CottonGen database.^29^ Coding sequences (CDS) of *GhJAZ2-A* and *GhJAZ2-D* were aligned using NCBI BLAST^30^ to identify consensus regions for designing a single gRNA targeting both genomes of *Gossypium hirsutum* L. Guide-RNA oligos (Forward oligo: 5′-TTTCCCGGCCGACAAAGCTA-3′; Reverse oligo: 5′-TAGCTTTGTCGGCCGGGAAA-3′) targeting the *GhJAZ2* gene were designed using CHOPCHOP v3.^31^ The oligos were commercially synthesized as single strands with ATTG and AAAC adaptors added to the 5′ ends, respectively. Potential off-target sites were also assessed using Cas-OFFinder.^32^

### 2.2. CRISPR/Cas9 Vector Construction

The plant binary vector pHSE401 (Addgene plasmid #62201) was used for cloning and expression of CRISPR reagents. The pHSE401 vector contained two BsaI restriction sites used for gRNA cloning. It was digested with BsaI (NEB, Cat. No. R3733S) and purified by gel extraction using the GeneJET Gel Extraction Kit (Thermo Fisher Scientific, Cat. No. K0691). Forward and reverse oligos were first annealed by combining 9 µL of each 100-fold diluted oligo (100 µM) with 10x NEBuffer 2.1 (NEB, Cat. No. B7202) in a total reaction volume of 20 µL. The mixture was heated to 95°C for 5 min, then gradually cooled to 25°C by incubating at room temperature for 30 min. These annealed oligos were then ligated into a BsaI-digested pHSE401 vector using T4 DNA ligase (Thermo Fisher Scientific, Cat. No. EL0011). Positive clones were verified by Sanger sequencing using MSQ-F primer (Table S1). The constructed vector (pHSE401-gRNA) was transformed into *Agrobacterium tumefaciens* strain GV3101 for cotton transformation.

**Table 1.** Germination rate and transformation efficiency in T_1_ generation.

T_0_ transformant	No. of seeds harvested	No. of seeds germinated (germination rate)	No. of hygro-resistant plants	No. of PCR-positive plants	Transformation efficiency
P_1_	–	–	–	–	–
P_2_	29	13 (44.82%)	0	0	0
P_3_	99	34 (34.34%)	3	3	8.82%
P_4_	61	10 (16.39%)	0	0	0
P_5_	125	38 (30.4%)	0	0	0
P_6_	75	40 (53.33%)	2	0	0
P_7_	87	28 (32.18%)	0	0	0
P_8_	42	11 (26.19%)	0	0	0
P_9_	63	4 (6.34%)	0	0	0
P_10_	112	0 (0%)	0	0	0
P_11_	–	–	–	–	–
P_12_	90	2 (2.22%)	0	0	0
P_13_	29	1 (3.44%)	0	0	0
P_14_	86	12 (13.95%)	0	0	0
P_15_	113	58 (51.32%)	3	3	5.17%
P_16_	97	64 (65.97%)	4	3	4.68%
P_17_	–	–	–	–	–
P_18_	109	33 (30.27%)	3	3	9.09%
P_19_	71	5 (7.04%)	0	0	0
P_20_	67	15 (22.38%)	0	0	0
P_21_	18	18 (100%)	0	0	0
P_22_	100	8 (8%)	0	0	0
P_23_	–	–	–	–	–
P_24_	89	15 (16.85%)	0	0	0
P_25_	78	16 (20.51%)	0	0	0
P_26_	–	–	–	–	–
**Total**	**16,50**	**425 (25.75%)**	**15**	**12**	**2.82%**

“–” indicates that data are unavailable because the plants died at early developmental stages and failed to produce seeds.

### 2.3. *In Planta* Agrobacterium-Mediated Transformation

A modified shoot apical meristem-targeted *in planta* transformation strategy was employed ^25–28^ to transform Bt cultivar CKC-3. CKC-3 is a commercially cultivated transgenic cotton variety carrying insect and herbicide resistance genes (Cry1Ac + Cry2A + GTG).^33^ It was selected for its compatibility with transformation protocols ^34,35^ and its established performance in terms of yield and fiber quality (fiber length of 26 mm and lint percentage of 35%). Acid-delinted seeds were rinsed under running tap water and sterilized in 0.1% (w/v) mercuric chloride (HgCl_2,_ Sigma-Aldrich) with agitation on an orbital shaker at 90 rpm for 10 min, followed by 3–4 washes with autoclaved distilled water. Surface-sterilized seeds were soaked in sterile water for 2 h and sown into sterile cups containing autoclaved peat moss. Seeds were then allowed to germinate and grow in a growth chamber at 30°C, 70% relative humidity, and a 16/8 h photoperiod cycle for 4–7 days until full cotyledon expansion. A single colony of *A. tumefaciens* strain GV3101 carrying the pHSE401-gNRA construct was cultured in YEP broth supplemented with antibiotics at 28°C and 220 rpm overnight in the dark. The culture was centrifuged at 6000 rpm for 5 min, and the pellet was resuspended in an inoculation medium (10% sucrose (PhytoTech), 0.05% Silwet L-77 (PhytoTech), and 200 µM acetosyringone (PhytoTech)) to obtain an OD_600_ of 2.0. One of two fully expanded cotyledons of each germinated seed was carefully removed at the base of the stem using a sterile scalpel, exposing the shoot apex, which was placed between the remaining cotyledons. A 20 µL of previously prepared Agrobacterium suspension was dripped on the exposed shoot apex using a micropipette, transferring Agrobacterium cells to the injured shoot apex. After the infection of seedlings with Agrobacterium suspension on the first day, the same Agrobacterium inoculation procedure was repeated on the same seedlings on the second and third days. The seedlings were incubated in the dark at 28°C during these three days. On the fourth day, the wounded shoot apex of seedlings was washed three times with a cefotaxime solution (conc. 500 mg/L). Then, seedlings were transferred to the light for 2 weeks in a growth chamber and watered at regular intervals. After 2 weeks, the seedlings were transferred to new, larger plastic pots with soil in the glasshouse to grow into mature plants and produce T_1_ transformant seeds.

### 2.4. Screening of Primary Transformants for the T-DNA Integration

Putative transformants were screened via PCR to confirm the presence of T-DNA in the genome. Genomic DNA was extracted from both wild-type and Agrobacterium-inoculated plants that survived infection, using a modified CTAB protocol reported by Ali et al.^36^ Approx. 200 mg of leaf tissue taken from one of the true leaves was ground in 600 μL of pre-warmed extraction buffer (100 mM Tris-HCl (pH 8.0; Sigma-Aldrich), 20 mM ethylenediaminetetraacetic acid (EDTA, Sigma-Aldrich), 1.4 M sodium chloride (NaCl, Sigma-Aldrich), 0.5 M glucose (Sigma-Aldrich), 2% w/v polyvinylpyrrolidone (PVP, Sigma-Aldrich), 1.0% w/v sodium dodecyl sulfate (SDS, Sigma-Aldrich), 2% w/v cetyltrimethylammonium bromide (CTAB, Sigma-Aldrich), and 0.5% v/v 2-mercaptoethanol (BME, Sigma-Aldrich), and nuclease-free water) using a sterile mortar and pestle. The homogenate was transferred to a 1.5 mL eppendorf vial, vortexed for 30 sec, and incubated at 65°C for 30 min with intermittent shaking. Then, an equal volume of chloroform: isoamyl alcohol (24:1, Sigma-Aldrich) was added, mixed by inversion, and centrifuged at 14,000 rpm for 15 min at 4°C. The aqueous phase was carefully transferred to a new vial, mixed with an equal volume of chilled 2-propanol (Sigma-Aldrich), and incubated at −20°C for 15 min to precipitate DNA. The sample was then centrifuged at 12,000 rpm for 10 min at 4°C. The resulting pellet was washed with 250 μL of 70% ethanol (Sigma-Aldrich), centrifuged at 10,000 rpm for 5 min at 4°C, and air-dried. Finally, the DNA was resuspended in 50 μL of nuclease-free water and incubated at 50°C for 5 min. The purity and concentration of the extracted DNA were assessed using a NanoDrop^TM^ 2000 Spectrophotometer (Thermo Fisher Scientific).

Following genomic DNA extraction, PCR was performed to amplify the Cas9 gene using gene-specific primers listed in Table S1. Each 10 μL of PCR reaction mixture contained 1× DreamTaq Green PCR Master Mix (Thermo Fisher Scientific, Cat. No. K1081), 0.5 μM each of 10 μM primers, 100 ng of template DNA, and nuclease-free water. The PCR setup also included negative and positive controls. PCR amplification was carried out using a Bio-Rad T100 thermal cycler under the following conditions: initial denaturation at 95°C for 3 min; 35 cycles of denaturation at 95°C for 30 s, annealing at 60°C for 30 s, and extension at 72°C for 30 s. Then, the PCR product, along with a 100 bp DNA ladder (Thermo Fisher Scientific, Cat. No. SM0241), was electrophoresed on an ethidium bromide-stained 2.0% agarose gel (1× TAE buffer) at 80 V for 40 min, and visualized using a ChemiDoc™ MP Imaging System (Bio-Rad).

### 2.5. Screening of Stable T_1_ Transformants

Cotton seeds were harvested from mature primary transformants grown under controlled glasshouse conditions. Seeds were delinted using concentrated sulfuric acid (H_2_SO_4_, Sigma-Aldrich), rinsed 3–4 times with distilled water, and soaked for 2 h. Both T_1_ and control seeds were then sown in autoclaved peat moss in plastic trays and germinated in a growth chamber (30°C, 70% RH, 16/8 h photoperiod) for 4–7 days. T_1_ transformants were initially screened using a hygromycin-resistance assay ^37,38^ followed by PCR confirmation.

#### 2.5.1. Hygromycin Resistance Assays

To determine the effective hygromycin dose, preliminary trial assays were conducted on wild-type cotton plants with concentrations of 20, 40, 60, and 80 mg/L. The lethal dose causing 80–90% mortality in wild-type plants was selected for screening transformed plants. At the fourth leaf stage, cotton seedlings were sprayed with a standardized dose on three alternative days and scored for resistance or sensitivity two weeks post-treatment. An untreated control group was also maintained. To further eliminate any remaining non-transformed seedlings, a leaf painting assay ^39^ was performed at the sixth-leaf stage. Expanded leaves from the top, middle, and lower canopy were brushed with the same hygromycin dose. Cotton plants showing necrosis after one week were discarded. Surviving plants were subjected to further molecular analyses.

#### 2.5.2. Genomic DNA Extraction and PCR Assay

Genomic DNA was extracted from the leaf tissue of hygromycin-resistant cotton plants using the CTAB method described above. Purity and concentration of extracted DNA samples were assessed using a NanoDrop™ 2000 Spectrophotometer (Thermo Fisher Scientific). To amplify the Cas9 gene, PCR was performed using gene-specific primers (Table S1) in a 10 µL reaction containing 100 ng template DNA, 1× of DreamTaq Green PCR Master Mix (Thermo Fisher Scientific, Cat. No. K1081), 0.5 µM of each primer, and nuclease-free water. All the controls were also included. PCR conditions were: initial denaturation at 95°C for 3 min, followed by 35 cycles of 95°C for 30 s, 60°C for 30 s, and 72°C for 30 s. Amplified products were resolved on a 2.0% EtBr-stained agarose gel (1× TAE buffer) alongside a 1 kb Plus DNA ladder (NEB, Cat. No. N3200S), electrophoresed at 80 V for 40 min, and visualized using a ChemiDoc™ MP Imaging System (Bio-Rad).

### 2.6. Quantification of Cas9 mRNA in Transformants

The accumulation of the Cas9 gene transcript in putative transformants was assessed by quantitative RT-qPCR. Total RNA was extracted from wild-type and transgenic cotton plants using the TRIzol protocol. A 100 mg of leaf tissue was ground in liquid nitrogen, and 1 mL of TRIzol (Invitrogen, Cat. No. 15,596,026) was added. The homogenate was transferred to a 1.5 mL eppendorf vial and incubated for 5 min at room temperature. Next, 200 µL of chloroform (Sigma-Aldrich) was added, and the vial was shaken vigorously by hand for 15 s, and then incubated for an additional 5 min. Samples were centrifuged at 12,000 rpm for 15 min at 4°C, and the aqueous phase was transferred to a new vial. RNA was precipitated by adding 500 µL of 2-propanol (Sigma-Aldrich) and incubating at room temperature for 15 min, followed by centrifugation at 12,000 rpm for 10 min at 4°C. The resulting pellet was washed with 1 mL of 70% ethanol (Sigma-Aldrich), centrifuged at 10,000 rpm for 5 min at 4°C, air-dried, and dissolved in 50 µL of RNase-free water. RNA quantity and quality were assessed using a NanoDrop™ 2000 Spectrophotometer (Thermo Fisher Scientific). cDNA synthesis from extracted RNA was performed using the RevertAid First Strand cDNA Synthesis Kit (Thermo Fisher Scientific, Cat. No. K1621).

For quantitative PCR (qPCR), we used the Bio-Rad C1000 Touch thermal cycler in a reaction mixture containing 0.25 µM of each Cas9 gene-specific primer (Table S1), 1× of Maxima SYBR Green Master Mix (Thermo Fisher Scientific, Cat. No. K0221), 100 ng of diluted cDNA template, and nuclease-free water to make up the final volume of 20 µL. The glyceraldehyde 3-phosphate dehydrogenase gene (*GhGAPDH*) was amplified using a gene-specific primer pair listed in Table S1 as an internal control with a PCR profile of 10 min at 95°C, followed by 40 cycles of denaturation at 95°C for 30 s and annealing at 60°C for 30 s. Ct values for target (Cas9) and reference (*GhGAPDH*) genes were recorded. Primer specificity was checked by melting curve analysis. Relative gene expression was determined by the 2^−ΔΔCt^ method.^40^ For each biological sample, the ΔCt value was computed as the difference between the Ct value of Cas9 and *GhGAPDH*. The ΔΔCt value was then calculated by normalizing each transgenic line to the wild-type control (calibrator). Relative expression levels were subsequently presented as 2^−ΔΔCt^. Three technical replicates were performed for each biological sample.

### 2.7. Validation of *GhJAZ2*-Targeted Editing Through Sanger Sequencing

The Sanger sequencing of the gRNA target site in the *GhJAZ2* gene was performed to evaluate the nature of induced mutations in transformants. The PCR was conducted in a 50 µL reaction volume using 2× Phusion High-Fidelity PCR Master Mix (Thermo Fisher Scientific, Cat. No. F531L) and *GhJAZ2* gene-specific primers (listed in Table S1). PCR conditions included an initial denaturation at 98°C for 3 min, followed by 35 cycles of 98°C for 30 s, 62°C for 30 s, and 72°C for 30 s, with a final extension at 72°C for 5 min. Amplicons were sent for sequencing to Synbio Technologies Company (New Jersey, USA). The resulting chromatograms were assessed using the DECODR v3.0 tool ^41^ to identify mutation patterns.

### 2.8. *GhJAZ2* Gene Expression Analysis Using RT-qPCR

‎To conduct a gene expression analysis of *GhJAZ2*, total RNA was purified from the ovules of edited and wild-type cotton lines at the primary stage of fiber development using the TRIzol-based procedure described earlier. Then, cDNA synthesis was done using the RevertAid First Strand cDNA Synthesis Kit (Thermo Fisher Scientific, Cat. No. K1621). The RT-qPCR reaction mixture included 50 μL Maxima SYBR Green Master Mix (Thermo Fisher Scientific, Cat. No. K0221), 0.5 μL of forward and reverse primer (10 μm) specific to the *GhJAZ2* gene, 1 μL of cDNA, and 30 μL of nuclease-free water to bring the final volume to 10 μL. The RT-qPCR cycling conditions, consisting of 95°C for 10 min, 40 cycles of 95°C/30 s and 62°C/30 s, with a final melting curve analysis, were applied. The RT-qPCR was performed in triplicate. Target gene expression was normalized using *GhGAPDH* as a reference gene. The double delta CT method was used to determine relative expression ^40^ and was calculated as a fold change relative to the control.

### 2.9. Fiber Analysis

Fully matured cotton bolls were harvested individually from *GhJAZ2*-edited plants along with wild-type control plants. The bolls were ginned using a laboratory-type roller gin. Three main fiber quality traits, including fiber length (mm), micronaire value (μg/in), and fiber strength (g tex −1), were measured using a high-volume instrument (USTER, HVI-1000). The lint percentage (%) was also calculated by weighing the lint from 100 seeds with fibers attached from each plant.^10^

For each plant, 20–25 bolls were collected from the upper, middle, and lower canopy positions and pooled to obtain a representative sample. Three independent biological replicates were used for each genotype. All data were subjected to one-way ANOVA using Minitab 17 statistical software.^42^

## 3. Results

### 3.1. Guide-RNA and CRISPR/Cas9 Vector

**Figure 1.**
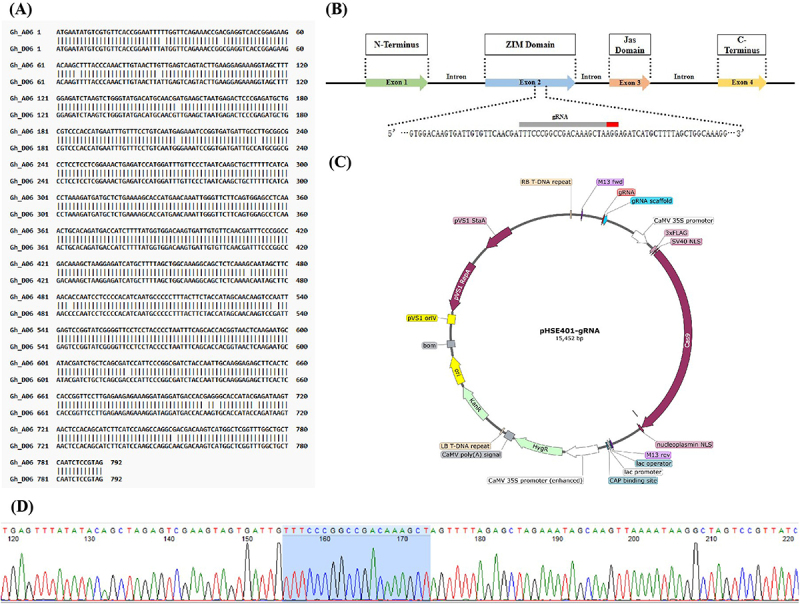
Construction and validation of the pHSE401-gRNA construct targeting the *GhJAZ2* gene. (A) Pairwise sequence alignment between the CDS of two JAZ2 homologs, Gh_A06 and Gh_D06, from the at and Dt sub-genomes, respectively, was generated using NCBI blast. Identical nucleotides are depicted by vertical bars (|), and numbers on the right show nucleotide positions. (B) The gene structure is shown with the annotated transcript with the consensus sequence from chromosomes A06 and D06 below. The gRNA target site is marked in blue; the pam (5′-NGG-3′) is indicated by a black bar within exon 2. (C) A map of construct pHSE401-gRNA. (D) Sanger sequencing result of the ligated product, in which the ligated gRNA is highlighted with blue colour.

To construct a CRISPR/Cas9-based vector containing a gRNA targeting the *GhJAZ2* gene in cotton, a single gRNA (guide sequence with protospacer-adjacent motif (PAM, in bold): 5′-TTTCCCGGCCGACAAAGCTA**AGG**-3′) was designed on the sense strand after generating a consensus sequence of the *GhJAZ2*. Because pairwise alignment between coding sequences (CDS) of both *GhJAZ2* homologs revealed 98% sequence identity, as shown in Figure 1A, enabling the simultaneous targeting of both *GhJAZ2-A* and *GhJAZ2-D*. The gRNA targets the ZIM domain located on exon 2 of both the A and D sub-genomes (Figure 1B). The selection of the target site (ZIM domain) in *GhJAZ2* was based on our previous in silico study, which showed that the ZIM domain of the GhJAZ2 protein is crucial for interactions between cotton GhJAZ2 and GhWD40, GhMYB25-like, GhGL1, GhMYC2, and GhJI1 proteins in the JA signaling to modulate the fiber initiation.^43^ The gRNA used in this study had an efficiency score of 56.07, predicted by the web-based CHOPCHOP tool. However, experimental results did not match this prediction. Genome-wide off-target analysis with Cas-OFFinder found no potential off-target sites for the designed gRNA, indicating high specificity for the intended target sequence. For gRNA cloning, the CRISPR/Cas9 vector (pHSE401) used in our study is based on the pCAMBIA backbone and includes an sgRNA module (Figure 1C). It requires only the Type IIs restriction enzyme BsaI for single-step cloning, eliminating the need for additional restriction enzymes. The construct expresses a maize codon-optimized Cas9 driven by the Cauliflower Mosaic Virus 35S (CaMV35S) promoter and a gRNA under the Arabidopsis U6 (AtU6) promoter. Sequencing of the construct confirmed not only the successful ligation of the gRNA but also its correct integration into the pHSE401 vector at the intended ligation site (Figure 1D).

### 3.2. *In Planta*Agrobacterium-Mediated Transformation in G. hirsutum L. with Integration of T-DNA in T_0_ Transformants

**Figure 2.**
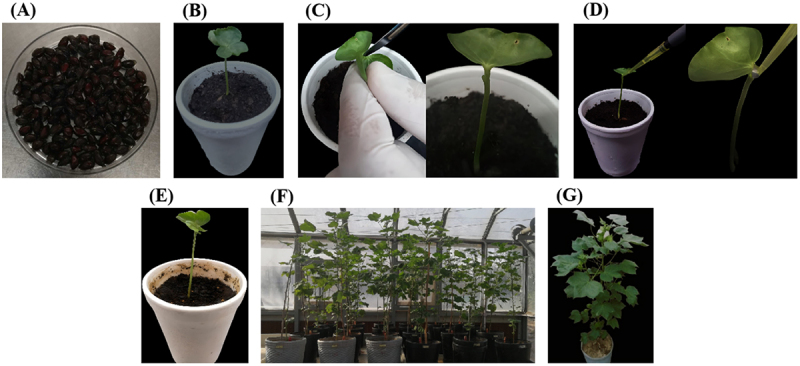
Illustration of the shoot apical meristem-targeted *in planta* transformation procedure used in the current study. (A) delinted and sterilized seeds of cotton (*G. hirsutum* L.: cv. CKC-3). (B) cotton seedling with fully expanded cotyledons before inoculation. (C) Removal of one cotyledon from the seedling with a sterile scalpel and exposure of the shoot apical meristem cells. (D) Dripping of Agrobacterium suspension on the wounded shoot apex of the seedling through a micropipette. (E) Two weeks old inoculated cotton plant. (F) Two months old transgenic cotton plants in the glasshouse. (G) A mature transgenic cotton plant with flowers and bolls.

For *in planta* transformation, the targeted shoot apical meristem (Figure 2), 250 delinted seeds of G. hirsutum L. cv CKC-3 (Bt cotton) were initially sterilized and sown in sterile plastic cups containing autoclaved peat moss. Then, one of the two fully expanded cotyledons of 4–7 days old cotton seedlings was removed using a sterile scalpel. Agrobacterium infection was repeated three times at one-day intervals. In total, 150 out of 250 seeds germinated successfully, showing a germination rate of 60%. The number of seedlings infected was equal to the number of seeds germinated. Among these, 93 seedlings survived post-infection, indicating a survival rate of 62%. 26 out of the 93 plantlets tested positive via PCR-based screening, resulting in a transformation efficiency of 17.33%. As shown in Figure 4A, all 26 out of 93 plantlets produced the expected 199 bp amplicon of the Cas9 gene, providing evidence for successful T-DNA integration in these putatively transformed plantlets. Chimerism was observed among the PCR-positive T_0_ plants. PCR screening of individual cotton branches indicated variable presence of the Cas9 transgene, with only a subset of plants showing positive amplification signals (Figure S1). Specifically, only 15.62% of branches were positive, while 84.32% tested negative, further confirming the chimeric nature of the primary transformants. The PCR-positive plants were selected as putative transformants and transferred to pots containing soil in the glasshouse, where they attained maturity, flowered, and produced seeds. However, the growth rate in these plants was slower than that of the wild-type.

### 3.3. Confirmation of Stable T_1_ Transformants by Hygromycin-Resistant Assays and PCR

**Figure 3.**
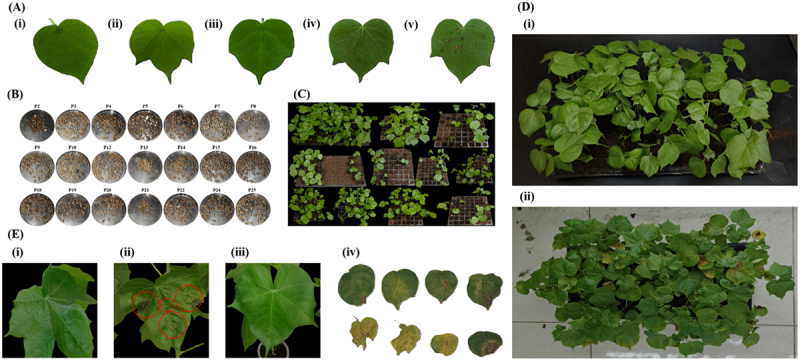
Hygromycin-resistant assays for screening T_1_ transformants. (A) Determination of lethal hygromycin dose. Variation in wild-type plant leaf response to different hygromycin doses **(i)** non-sprayed control (normal), **(ii)** 20 mg/L (no necrotic patches), **(iii)** 40 mg/L (no necrotic patches), **(iv)** 60 mg/L (fewer necrotic patches), **(v)** 80 mg/L (more necrotic patches). (B) Illustration of all the delinted T_1_ seeds harvested from independent primary transformants. (C) Illustration of seedlings germinated from all the T_1_ seeds after 14 days. (D) Hygromycin spray assay (70 mg/L) on the fourth leaf stage T_1_ seedlings, (I) T_1_ seedlings before hygromycin spray; healthy and green T_1_ seedlings, **(ii)** T_1_ seedlings after hygromycin spray; the non-transformed T_1_ seedlings with localized chlorosis and necrotic patches on their leaves and transformed T_1_ seedlings with green and healthy leaves. (E) Leaf painting assay with hygromycin solution (70 mg/L) on sixth leaf stage T_1_ plants, **(i)** green and healthy wild-type plant leaf without hygromycin application, **(ii)** wild-type (control) plant leaf with necrotic patches (red encircled) after hygromycin application, **(iii)** green and healthy transformed T_1_ plant leaf after hygromycin application, **(iv)** Different non-transformed T_1_ plants leaves with chlorosis and necrotic patches after hygromycin application.

A total of 16,50 seeds were harvested from 21 out of 26 primary transformants, and all were sown (Figure 3B). Of these, only 425 seeds germinated, as shown in Figure 3C, resulting in a germination rate of 25.75%. The yield and germination rate of T_1_ seeds of each primary transformant are given in Table 1.

Antibiotic-based screening reduces escapes by increasing the stringency in identifying putative transformants.^28^ Hygromycin impedes plant cell growth by binding to the 30S ribosomal subunit, distorting plastid translation initiation and directly affecting the leaves, which conduct photosynthesis, absorption, and transpiration.^37,38^ To identify putative transformants resistant to hygromycin by hygro-resistant assays, the lethal dose of hygromycin to be used for screening T_1_ transformants was initially identified. After 7 days of hygromycin spray with different concentrations, the leaves were scored for necrosis. Concentrations of 20 mg/L and 40 mg/L of hygromycin (Figure 3A ii and **iii**) did not show observable effects. However, 60 mg/L hygromycin exhibited necrotic spots on the leaf (Figure 3A iv). With further increase in the hygromycin concentration, a severe necrosis of the leaf was shown (Figure 3A v). In our results, the non-sprayed control devoid of hygromycin showed a normal phenotype with no symptoms (Figure 3A i). Based on the response of the wild-type leaves, 70 mg/L hygromycin was considered an optimal dose to confirm hygromycin resistance in putative T_1_ transformants. False-positive plants would result from a hygromycin concentration less than optimal, whereas true transgenic plants may die from a high concentration.

At the fourth leaf stage (approx. 10–14 days after germination), the 425 T_1_ seedlings were sprayed with 70 mg/L hygromycin solution for preliminary identification. It was observed that after two weeks of spraying, 390 (91.76%) T_1_ seedlings showed necrotic symptoms as well as turned yellow, showing susceptibility toward hygromycin (Figure 3D i and ii). However, T_1_ seedlings that survived by staying healthy and green were considered resistant to hygromycin. All surviving T_1_ seedlings with or without necrotic symptoms were transferred into plastic cups filled with autoclaved peat moss.

After one week of adaptation, healthy T_1_ plants at the sixth leaf stage were further screened by leaf painting using 70 mg/L hygromycin on respective plants to reduce the number of false positives (Figure 3E). After one week, T_1_ plants were observed for the presence or absence of necrotic patches and chlorosis. The leaf of the non-treated control plant remained green and healthy as hygromycin was not applied (Figure 3E i), and the leaves of control and non-transformed T_1_ plants were bleached and had necrotic patches as they were sensitive to hygromycin (Figure 3E ii and iv). However, the leaves of the hygromycin-resistant T_1_ plants remained healthy and green on hygromycin application, as shown in Figure 3E iii. About 15 (3.52%) T_1_ plants with hygromycin resistance were chosen due to the absence of any necrotic symptoms on treated leaves for PCR analysis based on two stringent hygromycin-resistant assays (Table 1). Thus, it’s shown that the hygromycin-based screening strategy was efficient and useful for offering strong discrimination between transgenic and non-transgenic plants.

**Figure 4.**
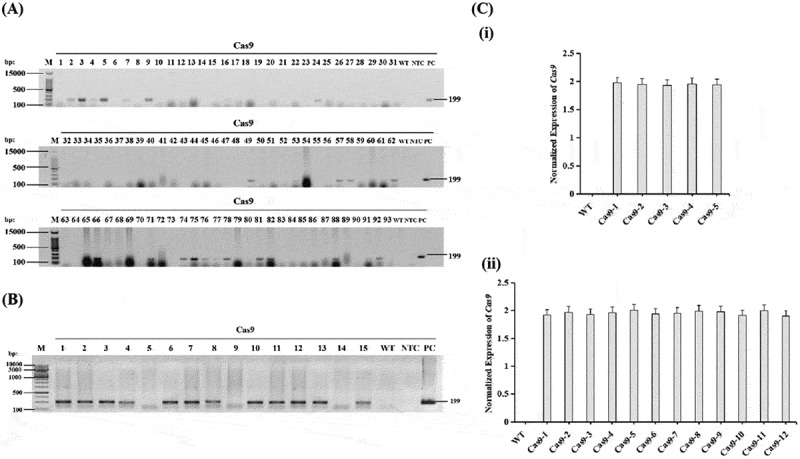
Molecular characterization of T-DNA integration and Cas9 expression in transformants. (A) PCR-based screening of the primary transformants. Lane M: 100 bp DNA ladder (Thermo Fisher Scientific); lanes 1–93: DNA of the recovered plants; lane WT: wild-type; lane NTC: No template control (water); lane PC: positive control (pHSE401 vector). (B) PCR analysis of the hygromycin-resistant T_1_ plants. Lane M: 1 kb plus DNA ladder (NEB); lanes 1–15: DNA of the hygromycin-resistant T_1_ plants; lane wt: wild-type; lane NTC: No template control; lane pc: positive control. (C) Relative expression of the Cas9 gene in T_0_ **(i)** and T_1_ **(ii)** transformants determined by RT-qPCR. Gene expression levels were normalized using the 2^⁻ΔΔCt^ method, with *GhGAPDH* as the internal reference gene and the wild-type sample as the calibrator. Error bars represent the standard error (SE) of three replicates.

To further confirm the T-DNA presence and stable integration in hygromycin-resistant T_1_ plants, PCR was conducted using Cas9 gene-specific primers in all 15 hygromycin-resistant T_1_ plants, which showed the presence of a 199 bp amplicon in 12 plants (Figure 4B). Wild-type DNA did not show any amplification in the PCR. PCR was repeated three times using newly extracted DNA from these transformed T_1_ plants to confirm whether these plants were truly transformed or not. A 199 bp PCR product was consistently amplified in these plants every time, with no such amplification observed in the wild-type cotton genomic DNA. Amplification of the 199 bp amplified fragment of the Cas9 gene in 12 (80%) plants out of 15 hygromycin-resistant T_1_ plants confirmed a stable integration of the T-DNA. Hence, the overall transformation efficiency was 2.82% in the T_1_ generation (Table 1).

In addition, the gene stability was observed in the T_1_ generation, as all the leaves of each T_1_ transformant were PCR-positive for the Cas9 gene (Figure S2). T_1_ plants confirmed as PCR-positive were transferred to large pots containing soil in the greenhouse and successfully produced seeds.

### 3.4. Molecular Confirmation of Cas9 Gene Expression in Transformants

RT-qPCR of Cas9 results demonstrated that it is present in T_0_ and T_1_ transformants, while no expression of Cas9 was observed in wild-type (non-transgenic) controls. As an internal reference, the *GhGAPDH* housekeeping gene exhibited stable expression across both wild-type and transgenic samples, validating its use for normalization. Quantitative assessment revealed that Cas9 expression was stably maintained among the transformants, showing only minimal inter-line variability. The normalized expression values for T_0_ and T_1_ transformants are shown in Figure 4C (i and ii), indicating sustained expression of the transgene across generations.

### 3.5. Sanger Sequencing Confirms Targeted Editing of the *GhJAZ2* Gene

To assess CRISPR/Cas9-induced insertions and deletions (indels) at the gRNA-targeted site of *GhJAZ2*, transformants carrying the Cas9 gene were analyzed by Sanger sequencing. High-fidelity PCR was performed on genomic DNA from these plants using site-specific primers to amplify the targeted *GhJAZ2* region. PCR amplicons were then subjected to Sanger sequencing with the same primers.

**Figure 5.**
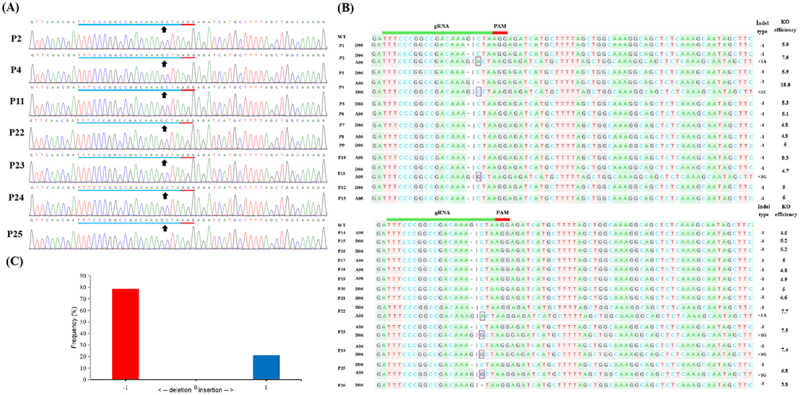
**Editing profile of primary transformants based on Sanger sequencing. (a)**Chromatograms and representative sequences identified by Sanger sequencing from edited plants. The black arrows represent the cleavage sites. **(b)** Sequence alignment to identify indel types of gRNA target sites in edited plants. The top one is a wild-type (wt) sequence from the *GhJAZ2-A/D* gene. The indel type and its position on the A_t_ or D_t_ sub-genome in each edited plant are listed on the right and left, respectively. “P” indicates an edited plant, “A06” indicates chromosome 06 on the A_t_ sub-genome, “D06” indicates chromosome 06 on the D_t_ sub-genome, “+” indicates base insertion, and “−” indicates base deletion. Deletions and insertions are denoted by dashes and square letters in sequences, respectively. Knockout (KO) efficiency (%) in all mutants was predicted by KO analysis (Pearson’s r correlation (R2) = 0.97) of Sanger sequencing traces using DECODR. **(c)** Frequency of different indels at the predicted *GhJAZ2* target site in 26 edited T_0_ plants.

**Figure 6.**
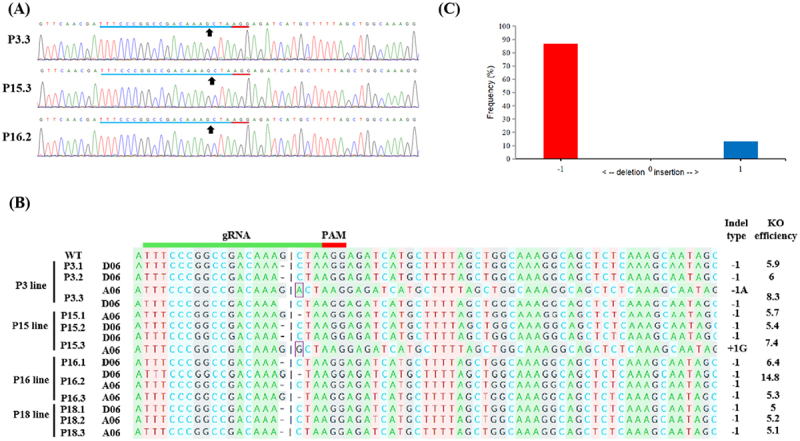
Editing profile of T_1_ lines based on Sanger sequencing. (A) Chromatograms and representative sequences identified by Sanger sequencing from edited T_1_ plants. The black arrows represent the cleavage sites. (B) Sequence alignment to identify indel types of gRNA target sites in edited T_1_ plants. The top one is a wild-type (wt) sequence from the *GhJAZ2-A/D* gene. The indel type and its position on the A_t_ or D_t_ sub-genome in each edited plant are listed on the right and left, respectively. “P” indicates an edited plant, “A06” indicates chromosome 06 on the A_t_ sub-genome, “D06” indicates chromosome 06 on the D_t_ sub-genome, “+” indicates base insertion, and “−” indicates base deletion. Deletions and insertions are denoted by dashes and square letters in sequences, respectively. KO efficiency (%) in all mutants was predicted by KO analysis (Pearson’s r correlation (R2) = 0.97) of Sanger sequencing traces using DECODR. **(c)** Frequency of different indels at the predicted *GhJAZ2* target site in 12 edited T_1_ plants.

Amplified products from all transformants, obtained using a common primer set, exhibited a distinct phenomenon in the sequencing chromatogram due to the tetraploid nature of cotton. As illustrated in Figures 5A and 6A, the standard sequencing chromatogram of these PCR fragments displayed single peaks extending up to the mutation regions of the gRNA target sequence. However, immediately beyond these mutation regions, multiple overlapping peaks emerged at each nucleotide position, complicating sequence resolution. A web-based tool, DECODR, was used to differentiate between these sorts of mutations. The various types of indels resolved using this web-based tool are shown in Figures 5B and 6B.

Sanger sequencing results revealed that all 26 primary transformants detected with indels at the target site. Among the 26 edited plants, 7 (P2, P4, P11, P22, P23, P24, and P25) were edited simultaneously in both the At and Dt sub-genomes, while the remaining plants were edited only in either the At or Dt sub-genome, as depicted in Figure 5B. The results from these editing sequences revealed that 26 out of 33 editing events were 1 bp nucleotide deletions (78.78%), while the remaining events were 1 bp nucleotide insertions (21.21%) (Figure 5C). Editing efficiency of the gRNA in edited primary transformants, calculated as the proportion of alleles per plant carrying mutations at the *GhJAZ2* target site, ranged from 3.8% to 18.8%.

In the T_1_ progeny, the 10 edited T_1_ plants (P3.1, P3.2, P3.3, P15.2, P15.3, P16.1, P16.2, P18.1, P18.2, and P18.3) kept the identical mutation of single nucleotide deletion as in their parents (P3, P15, P16, and P18), which are shown in Figure 6B. Interestingly, T_1_ progenies of these T_0_ plants also gained new mutations, including single-nucleotide insertions and deletions. One “A” insertion for the P3 progenies (P3.3), one “G” insertion and one nucleotide deletion for P15 progenies (P15.1 and P15.3), and one nucleotide deletion for P16 progenies (P16.2 and P16.3) were observed. These new indels may happen during the reproduction or development phases of T_1_ progenies. These findings confirmed that Cas9 and gRNA cassettes are effectively incorporated into meristematic L2 cells, which produce mutations in germline cells. Of the 15 editing events analyzed, 86.7% (13 events) were single-nucleotide deletions, and 13.3% (2 events) were single-nucleotide insertions (Figure 6C). Editing efficiency of the gRNA ranged from 5 to 14.8% in edited T_1_ progenies.

Overall sequencing analysis of T_0_ and T_1_ generations revealed that CRISPR/Cas9-induced mutations were predominantly single-nucleotide deletions (81.25%), while insertions accounted for 18.75% of the events. All of the indels were single-nucleotide deletions or insertions, as shown in Figures 5 and 6. Thus, 1 bp nucleotide indels in T_0_ and T_1_ plants were efficiently generated through CRISPR/Cas9-mediated genome editing in cotton. The mutation rate (the ratio of edited plants to the total number of transformants) was 100% in both T_0_ and T_1_ generations as determined by directly sequencing the PCR products of the *GhJAZ2* gene in each Cas9-positive plant. However, homozygous edited plants were not found, as indicated by sequencing analysis. These results show that the gRNA effectively targeted the *GhJAZ2* gene, guiding Cas9-mediated genome cleavage with high precision. This led to efficient edits in the target sequence, including nucleotide deletions and insertions in T_0_ and T_1_ plants.

### 3.6. Expression of *GhJAZ2* Gene in Edited and Wild-Type Lines

**Figure 7.**
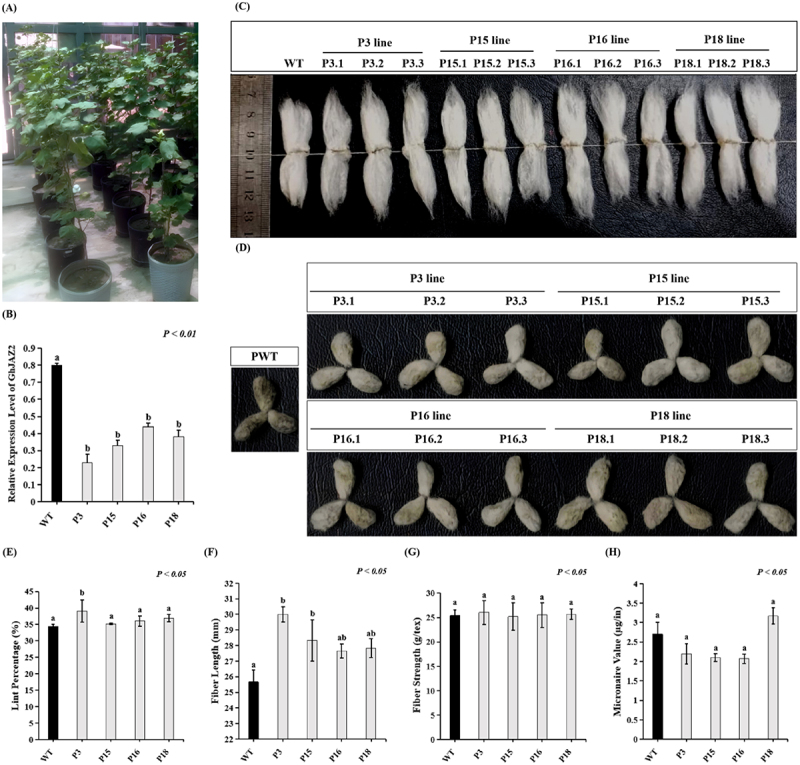
Analysis of fiber traits and *GhJAZ2* gene expression in wild-type line and *GhJAZ2*-edited lines. (A) mature wild-type line and edited lines with bolls. (B) Relative expression levels of *GhJAZ2* in ovules during the early stage of fiber development. Statistically significant differences are indicated by lowercase letters above the error bars, as determined by ANOVA followed by Dunnett’s post hoc test (*p < .01*). Data are presented as mean ±SE of 3 biological replicates (*n* = 3). (C) Mature lint fibers from wild-type and edited plants. Scale bar = 10 mm. (D) Phenotype of fiber-bearing seeds in the wild-type and edited plants. (E) Lint percentage of a hundred seeds with fibers attached from the control line and edited lines. (F) Length of fibers from the control line and edited lines. (G) Strength of fibers from the control line and edited lines. (H) Micronaire value of fibers from the control line and edited lines. Bars are indicative of the mean of three biological replicates ± SD. Statistical differences were assessed using a one-way ANOVA with Dunnett’s multiple comparison tests, where *p < .05* was considered significant.

The expression analysis of the *GhJAZ2* gene in edited cotton lines and the wild-type (WT) control was conducted using RT-qPCR. There was a considerable decrease (*p < .01*) in *GhJAZ2* transcript abundance in all the edited lines, with the lowest level of that transcript being in P3 (0.23 fold), P15 (0.33 fold), and P16 (0.44 fold) lines in comparison to the wild-type control (Figure 7B). Overall, these findings show that *GhJAZ2* expression in all edited lines was significantly reduced, indicating that *GhJAZ2* editing effectively suppressed its transcription during the initial phase of fiber development.

### 3.7. Fiber Quality and Yield in the *GhJAZ2*-Edited Lines

To investigate the phenotypic impact of CRISPR/Cas9-mediated editing of *GhJAZ2*, edited lines and wild-type (control) lines from the T_1_ generation were grown under controlled conditions in a greenhouse for detailed fiber analysis (Figure 7A). Fiber quality and yield parameters were assessed and compared to those of the control (Table 2).

**Table 2.** The lint percentage, fiber length, fiber strength, and micronaire value of the edited lines and the control line.

	Lint percentage(%)	Fiber length (mm)	Fiber strength (g/tex)	Micronaire value (µg/in)
WT	34.35 ± 0.67	25.67 ± 0.76	25.40 ± 1.20	2.70 ± 0.30
P3	39.07 ± 3.39^*^	30.00 ± 0.50^*^	26.03 ± 2.44	2.20 ± 0.26
P15	35.18 ± 0.28	28.33 ± 1.32^*^	25.20 ± 2.80	2.10 ± 0.10
P16	36.02 ± 1.64	27.67 ± 0.46^*^	25.50 ± 2.50	2.07 ± 0.12
P18	36.91 ± 1.11	27.83 ± 0.60^*^	25.70 ± 1.10	3.17 ± 0.21

* shows significant differences (*p < .05*) between wild-type and *GhJAZ2*-edited plants, and no star indicates a non-significant difference, as determined by Dunnett’s multiple comparison analysis. Data are presented as mean ± SD (standard deviation) from three independent biological replicates (*n* = 3) of each line. Each biological replicate corresponded to an individual plant. For each plant, 20–25 fully matured cotton bolls were harvested. Fibers from these bolls were pooled to make a representative sample for each biological replicate to measure fiber length, fiber strength, and micronaire value using HVI-1000. The lint percentage was determined by weighing the lint from 100 seeds with fibers attached from each plant.

Lint percentage, a crucial yield-associated trait in cotton, revealed a line-specific response to *GhJAZ2* editing (Figure 7D, E). The wild-type line showed an average of 34.35 ± 0.67%, whereas the edited line P3 exhibited a significant increase to 39.07 ± 3.39% (*p < .05*), corresponding to an improvement of approximately 13.74% over the control. In contrast, the lint percentages of lines P15 (35.18 ± 0.28%), P16 (36.02 ± 1.64%), and P18 (36.91 ± 1.11%) were not significantly different from the control, although a modest upward trend was observed.

Fiber length was consistently and significantly increased in all *GhJAZ2-*edited lines compared with the wild-type (Figure 7C,F). The control lines produced fibers with an average length of 25.67 ± 0.76 mm, whereas the edited lines displayed significantly longer fibers (*p < .05*), reaching 30.00 ± 0.50 mm in P3, 28.33 ± 1.32 mm in P15, 27.67 ± 0.46 mm in P16, and 27.83 ± 0.60 mm in P18. These results show that *GhJAZ2* editing has a strong positive effect on fiber elongation.

In contrast to fiber length, fiber strength did not differ significantly between the wild-type and any of the edited lines (Figure 7G). The wild-type fiber strength was 25.40 ± 1.20 g/tex, while the edited lines showed comparable values, including 26.03 ± 2.44 g/tex in P3, 25.20 ± 2.80 g/tex in P15, 25.50 ± 2.50 g/tex in P16, and 25.70 ± 1.10 g/tex in P18, indicating that *GhJAZ2* editing did not adversely affect this trait.

Micronaire values revealed variable, but largely non-significant variations among the edited lines (Figure 7H). The wild-type line showed a micronaire value of 2.70 ± 0.30, while P3 (2.20 ± 0.26), P15 (2.10 ± 0.10), and P16 (2.07 ± 0.12) exhibited lower values, indicating potentially finer fibers, but the observed difference was not significant. In contrast, line P18 exhibited a higher micronaire value of 3.17 ± 0.21 relative to the control. The difference, while suggesting a line-specific increase in micronaire value, was also not significant.

In conclusion, targeted editing of the *GhJAZ2* gene resulted in a robust and consistent enhancement in fiber length across all edited lines. However, improvements in lint percentage and micronaire value were line dependent. Notably, fiber strength remained unaffected, indicating that *GhJAZ2* editing increases key fiber quality traits without compromising mechanical properties.

## 4. Discussion

Cotton (*Gossypium hirsutum* L.) remains the world’s leading renewable natural fiber, accounting for approximately 40% of the annual fiber demand for the global textile and apparel industries.^1^ However, various biotic and abiotic stresses significantly compromise fiber quality and yield. To address these challenges, genome editing technologies have emerged as essential tools for developing cotton varieties with superior fiber quality and yield. In polyploid crops such as cotton, producing mutants via natural or artificial mutagenesis is challenging because of the functional redundancy of multiple gene copies.^15^ With recent advances in whole-genome sequencing, the CRISPR/Cas9 system has become a powerful and precise genome editing tool, enabling the targeted modification of specific genes in cotton in a time-efficient manner. To date, the CRISPR/Cas9 system has been used to generate precise mutations, including insertions, deletions, and base substitutions, primarily via the NHEJ pathway, leading to a limited yet steadily expanding range of gene-editing applications in upland cotton.^44^

The *GhJAZ2* gene is predominantly expressed during fiber initiation (0 DPA) in cotton floral and ovule tissues, where it functions as a key transcriptional repressor in the JA-signaling pathway to suppress the GhMYB25-like transcription factor activity.^10^ Overexpression of *GhJAZ2* has been shown to inhibit fiber initiation, suggesting that it negatively regulates both fiber initiation and elongation. Conversely, reduced *GhJAZ2* gene expression, coupled with increased JA concentration, promotes fiber initiation.^10^ These findings on the role of *GhJAZ2* in cotton largely relied on modulating gene expression through RNA interference (RNAi) and overexpression. The current study uses CRISPR/Cas9-based genome editing to precisely and simultaneously edit the *GhJAZ2* loci in both the A and D sub-genomes, providing a direct approach for producing beneficial alleles.

Agrobacterium-mediated transformation is the most common method for gene transfer in cotton due to its high efficiency, stable transgene integration, and consistent expression.^25^ Although alternatives like gene gun and pollen tube pathway exist, Agrobacterium, especially via somatic embryogenesis or shoot organogenesis, is preferred because it overcomes major limitations, including genotype dependence, complex tissue culture, and low regeneration efficiency,^22,45^ as well as elite transgenic lines often require extensive backcrossing. Hence, *in planta* transformation targeting the shoot apical meristem has emerged as an efficient, genotype-independent alternative.^25–28^ In our Agrobacterium-mediated *in plant* transformation strategy, we used shoot apical meristem (SAM) cells to transform elite cotton (cv. CKC-3) (Figure 2). During this process, T-DNA can be transferred into the genomes of many meristematic cells by *A. tumefaciens* that have not yet differentiated and are predestined to form specific organs. Traditional cotton transformation strategies, including somatic embryogenesis and shoot-tip organogenesis, typically exhibit low stable transformation efficiencies ranging from 0.2% to 2%.^25^ In contrast, our study achieved a stable transformation efficiency of 2.82% in the T_1_ generation, showing a notable improvement over conventional approaches and highlighting the potential of this strategy as a more efficient and reproducible method for producing stable transgenic lines in cotton. This efficiency also surpasses that reported in previous cotton shoot apical-based *in plant transformation* studies: Guo et al. ^26^ reported a T_1_ transformation efficiency of 0.125%, and Karthik et al. ^27^ reported 2.27%. These comparative results further highlighted the improved efficiency and reproducibility of the transformation strategy applied in this study. In addition, the inoculation medium, supplemented with Silwet L-77®, a high sucrose concentration, and acetosyringone, significantly improved transformation efficiency (17.33%) compared to the previously reported 6.89% at the T_0_ stage by Kalbande and Patil.^25^ Similar findings were reported in foxtail millet, where transformation efficiency improved using Silwet L-77® in combination with tobacco leaf extract, acetosyringone, and high levels of sucrose and glucose.^46^ Other Agrobacterium strains, such as LBA4044 and EHA105, can further intensify the efficiency of the *in planta* transformation strategy.^47^

Putative transformants obtained through *in planta* transformation have been reported as chimeric plants at the T_0_ stage in previous studies,^25–28^ which then produced stable transformants at the T_1_ generation. Consistent with previous findings, the primary transformants in our study also exhibited a chimeric pattern (Figure S1), but stable gene integration was successfully confirmed in the T_1_ progeny (Figure S2). Chimerism in primary transformants arises from the multicellular origin of shoots, where transformation is limited to a subset of cells within specific SAM layers (L1, L2, L3).^48^ This is increased by uneven *A. tumefaciens* infection and variable cellular competence, with transformation typically occurring in epidermal layer (L1) cells, and superficial layer (L2) and inner corpus region (L3) cells often remain untransformed, producing chimeric branches. The low detection rate in our study, with only 15.62% PCR-positive branches in primary transformants, suggests layer-specific transformation efficiency, likely influenced by greater accessibility of peripheral zone cells compared to central zone stem cells, as well as the developmental stage of the SAM at infection. To reduce chimerism, it is crucial to optimize transformation conditions by improving Agrobacterium infection efficiency, extending co-cultivation duration, and applying stronger and prolonged selection pressure, using a combination of antibiotics and herbicides during early regeneration to eliminate non-transformed cells. Additionally, targeting the meristem at the embryo or early seedling stage, when the SAM has few cells undergoing synchronous division. Such approaches can substantially improve the recovery of the non-chimeric T_0_ cotton plant.

Targeted genome editing in tetraploid cotton can produce diverse genotypes, including partial or complete knockouts, making their analysis complex. In several studies reported by Gao et al.,^14^ Zhu et al.,^49^ Li et al.,^50^ Li et al.,^17^ and Li et al.,^19^ Sanger sequencing was widely used due to its long read length, which enabled the detection of edits at multiple gRNA sites and the differentiation of allelic variants via SNPs. While effective for identifying simple or chimeric mutations, traditional clone-based Sanger sequencing is costly and labor-intensive. To streamline analysis, the DECODR tool enables accurate, quantitative assessment of multi-allelic indels from a single Sanger trace, outperforming other tools such as TIDE, ICE, Cas-analyzer, HI-TOM, and CRISP-ID by detecting a broader range of indel types and sizes.^41,51,52^ In our study, all independent transformants from both generations showed indels at the target site, as resolved by Sanger sequencing and traces through DECODR, which suggests a mutation rate comparable to or higher than previously reported studies.^15,48,53,54^ Most indels in *GhJAZ2 A/D* were single-nucleotide deletions, consistent across T_0_ and T_1_ plants (Figure 5C; Figure 6C) and aligning with patterns reported in other plant species.^48,55,56^ Identical edits in T_1_ plants confirmed their heritability and origin in the L2 cell layer of the shoot apical meristem, which gives rise to germ cells. Additionally, new indels that were failed in the T_0_ generation were also generated in the T_1_ progeny at the target site, confirming that CRISPR/Cas9 maintained its ability to perform gene editing in the T1 progeny when the target sequence was wild-type. This suggests that a relatively high percentage of indels could be induced in L2 cells by SAM-targeted *in planta* transformation. All the edited plants have shown a heterozygous genotype. However, obtaining a homozygous genotype in the T_0_ and T_1_ generations is challenging in cotton.^23^ Nevertheless, these edits can be stably inherited across subsequent generations.^57,58^ It has enormous promise to obtain homozygous edited lines of the target gene in the next generation.

The gRNA editing efficiency and off-target effects are two critical parameters to consider when utilizing the CRISPR/Cas9 system.^59,60^ In the present study, all edited T_0_ and T_1_ plants exhibited successful edits, with editing efficiencies of the gRNA ranging from 3.8 to 18.8% (Figure 5B; Figure 6B). Although various gRNAs can target the same gene with differing efficiencies, the DNA sequence of the target site within the gRNA plays a critical role in determining overall editing efficiency.^57,61^ Previous studies have also reported that several factors influence both editing efficiency and target specificity, including the promoter driving gRNA expression, the gRNA sequence, and the transformation method employed.^14,23^ The vector (pHSE401) used in this study has demonstrated high efficiency and specificity in previous studies involving transgenic maize lines, Arabidopsis lines, and maize protoplasts.^62^ In most CRISPR/Cas9 applications, Cas9 and gRNA expression are typically driven by the CaMV35S and AtU6 promoters, respectively.^14,20,23,24,48,54,63^ Moreover, no potential off-target sites were detected via web-based tool (Cas-OFFinder) analysis, even when allowing for a single-nucleotide mismatch, indicating high specificity of the CRISPR/Cas9 system used in our study. Cas-OFFinder differs from other off-target prediction tools because it offers greater flexibility by not limiting the number of mismatches and by allowing variations in the PAM region recognized by Cas9.^32^ Several previous studies have also shown that the off-target effects of the CRISPR/Cas9 system are consistently minimal in plant species.^64,65^

In addition, RT-qPCR analysis in our study revealed a significant reduction in *GhJAZ2* mRNA level across all edited cotton lines (Figure 7), suggesting that targeted genome editing disrupted *GhJAZ2* transcription and may alleviate GhJAZ2-mediated repression in JA signaling during the early stage of fiber development.

Cotton fibers are classified into two types: lint, the spinnable and economically important fiber that initiates at or before anthesis and elongates to 25–35 mm in length, and fuzz, a much shorter fiber (approx. 5 mm) that emerges later (4–5 DPA), persists after ginning.^6^ In our fiber analysis, the most consistent finding was a highly significant increase in fiber length across all edited lines. The ~4–5 mm gain compared to the wild-type line represents a substantial improvement in fiber quality. According to a previous study, *GhJAZ2* overexpression resulted in shorter lint fibers.^10^ Editing *GhJAZ2* in our study likely disrupted its repression, leading to constitutive activation of JA-responsive genes associated with fiber development in the JA signaling pathway and promoting enhanced cell elongation in developing fibers. The final fiber yield is largely determined during the initiation stage by the number of ovule epidermal cells that differentiate into fiber initials. Although all ovule epidermal cells have the potential to initiate fibers, only about 25–30% typically succeed,^66^ indicating a major limitation for improvement of lint yield. Consistent with the importance of this stage, Hu et al. ^10^ reported that *GhJAZ2* overexpression lines exhibited significantly fewer fiber initials than controls and RNAi lines, as determined by SEM. In our study, several edited lines showed notable increases (up to 13.74%) in lint percentage compared to the wild-type line, which is a direct yield component. These results suggest that disruption of *GhJAZ2* function may promote a higher proportion of ovule epidermal cells to differentiate into fibers during the initiation stage by enabling more favorable expression of positive regulators involved in fiber initiation, thereby contributing to the observed increase in lint percentage. Although our greenhouse phenotyping provides an initial assessment of the effects of targeted genome edits on fiber-associated traits in cotton, it lacks natural variability in factors including temperature, light intensity, wind, and biotic and abiotic stresses, which are key drivers of genotype-by-environment interactions. Consequently, trait heritability may be overestimated under greenhouse conditions. Therefore, conclusions regarding agronomic performance will require further validation under field conditions across various environments and growing seasons.

In conclusion, this study highlights the potential of CRISPR/Cas9-mediated genome editing as a precise and effective tool for improving complex agronomic traits in cotton. The edited lines developed here provide valuable intermediate genetic resources from which transgene‑free and superior cotton cultivars can eventually be derived. To obtain such transgene-free edited lines, a segregation-based strategy is applied in subsequent generations.^15,67,68^ T_1_ cotton lines carrying the CRISPR/Cas9 construct are selfed to produce T_2_ progeny, in which Mendelian segregation allows the recovery of lines that have lost the integrated T-DNA while retaining the desired edits. These putative transgene-free lines are identified by PCR-based screening using transgene-specific primers, followed by locus-specific amplification and sequencing to confirm the target mutation. Transgene-free T_2_ lines are further advanced to the T_3_ generation to confirm stable inheritance of the edit and complete absence of T-DNA. Although this segregation screening has not yet been performed in this study, the breeding strategy outlined above provides a clear path toward developing transgene-free edited germplasm, which is essential for regulatory approval and public acceptance. Future work will therefore focus on isolating and characterizing such lines.

## Supplementary Material

Supplemental Material

## Data Availability

The raw Sanger sequencing data generated and analyzed in this study are available in the NCBI Sequence Read Archive (SRA) under BioProject accession number PRJNA1401863, with SRA accession numbers SRX31780088–SRX31780125.
