# Supplementary material for: CRISPR/Cas9-mediated editing of the GhJAZ2 gene improves fiber length and lint percentage in Gossypium hirsutum L
Source: GM Crops Food. 2026 Apr 19;17(1):2660546. doi: 10.1080/21645698.2026.2660546 (PMC13094215; doi:10.1080/21645698.2026.2660546)
Supplement: Supplemental Material [file KGMC_A_2660546_SM1186.docx]

**Supplementary Material**

**Supplementary Table S1.** List of primers used in this study

| **Primer** | **Sequence (5’-3’)** |
| --- | --- |
| MSQ-F | AATCTTCAAAAGGCCCCTGG |
| Cas9-F | AGCTCGTCTCCGACTTCAGGA |
| Cas9-R | TGCTCCGACTTGGCGATCAT |
| GhJAZ2.Seq-F | GATATGTTGCAGAGACTCCCGA |
| GhJAZ2.Seq-R | TCGCTGAGCAGATCGTATGC |
| GhGAPDH-F | TGATGCCAAGGCTGGAATTGCTT |
| GhGAPDH-R | GTGTCGGATCAAGTCGATAACACGG |
| GhJAZ2.qP-F | GCATACGATCTGCTCAGCGA |
| GhJAZ2.qP-R | AACCGAGCCATGACTTGTCG |

**
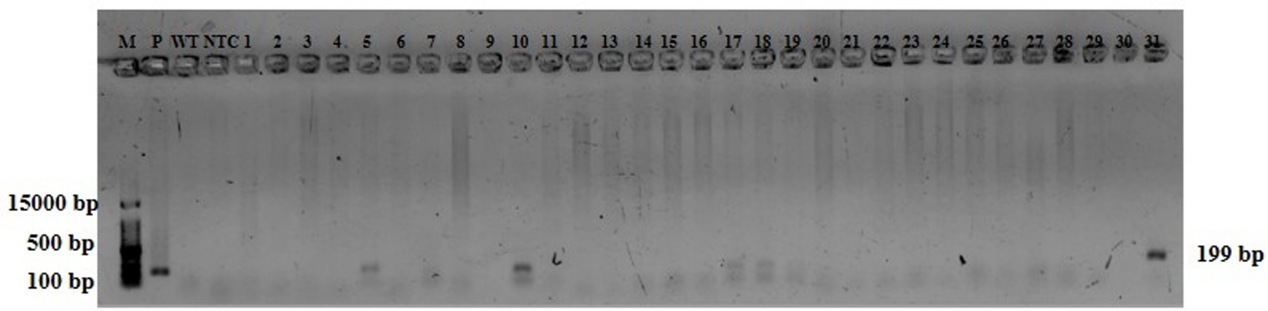
**

**Supplementary Figure S1.** Cas9 gene-specific PCR analysis of the isolated DNA from the branches of a primary transformant (T_0_). Lane M, 100 bp DNA ladder (Thermo Fisher Scientific); Lane P, Positive control (pHSE401 vector); Lane WT, wild-type; Lane NTC, No template control (water); Lanes 1-31, DNA of the each branch of a transformant (chimeric).

**
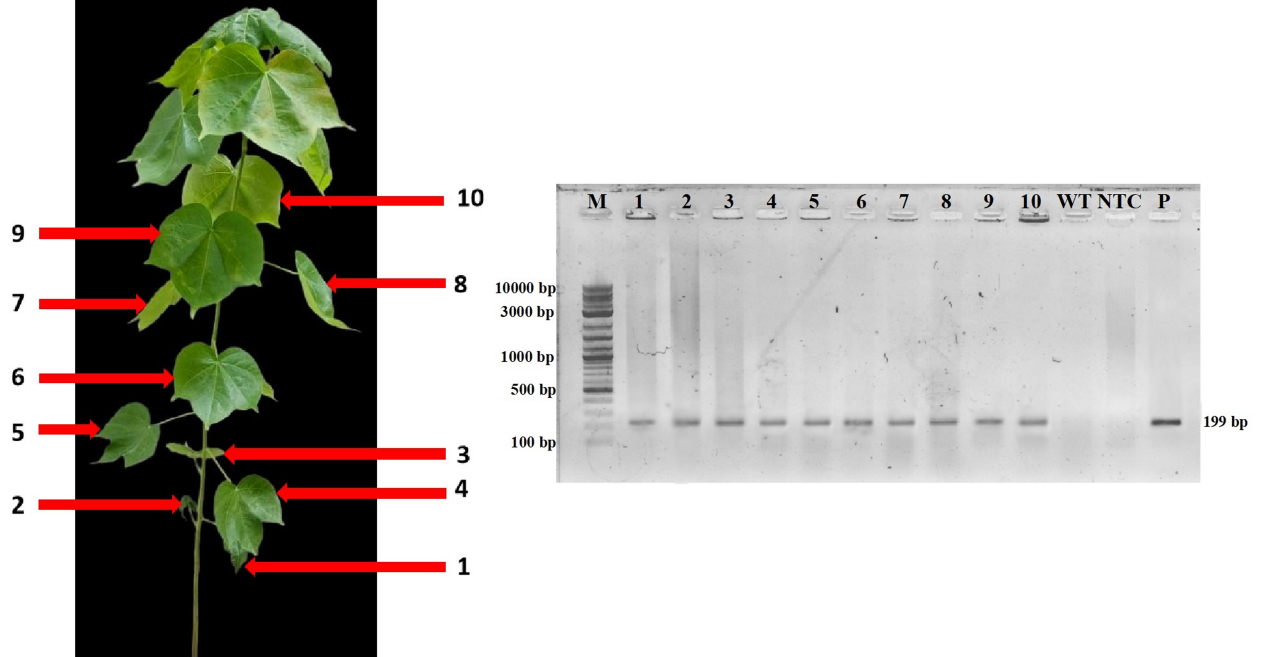
**

**Supplementary Figure S2.** Cas9 gene-specific PCR analysis of the extracted DNA from the leaves or branches of a single T_1_ transformant. Lane M, 1 kb plus DNA ladder (NEB); Lanes 1-10, DNA of each different leaf of a single transformant (stable); Lane WT, wild-type; Lane NTC, No template control (water); Lane P, Positive control (pHSE401 vector).

**
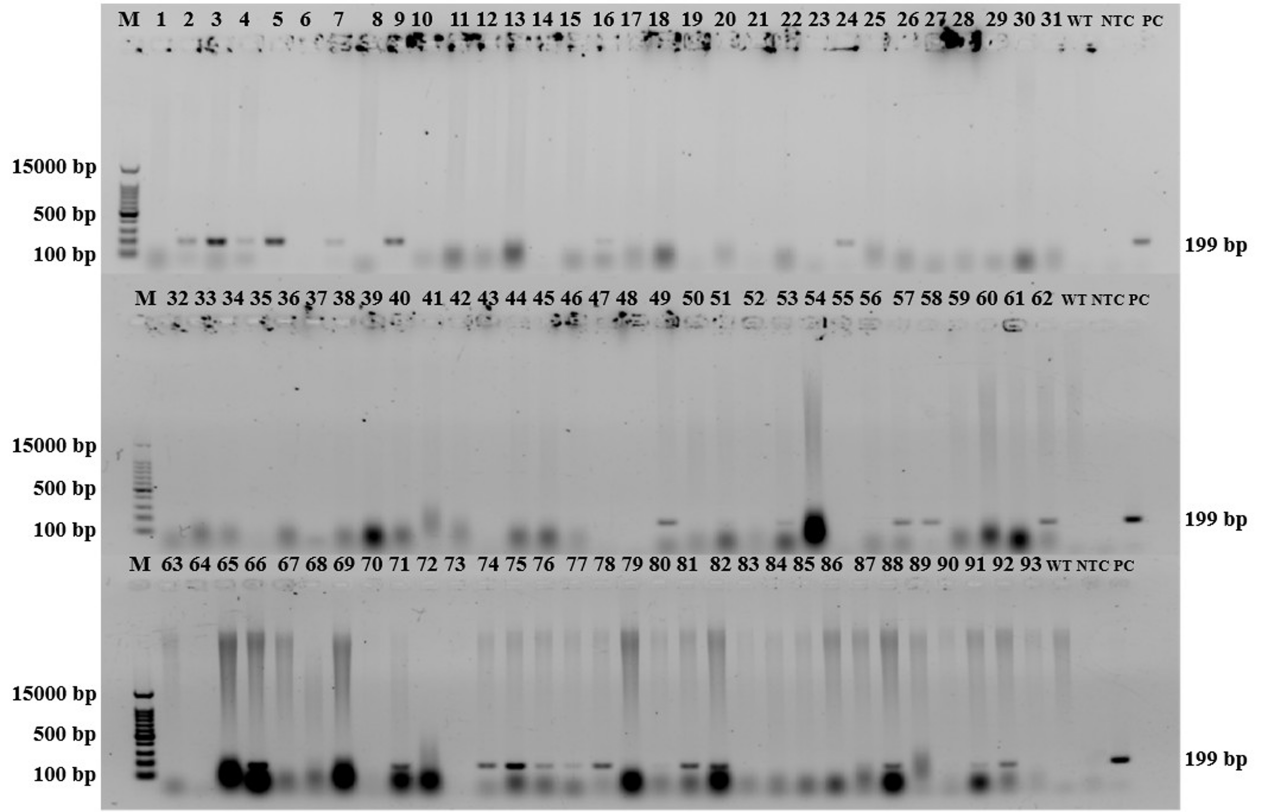
Supplementary Figure S3.** The original gel images shown in Figure 4A.


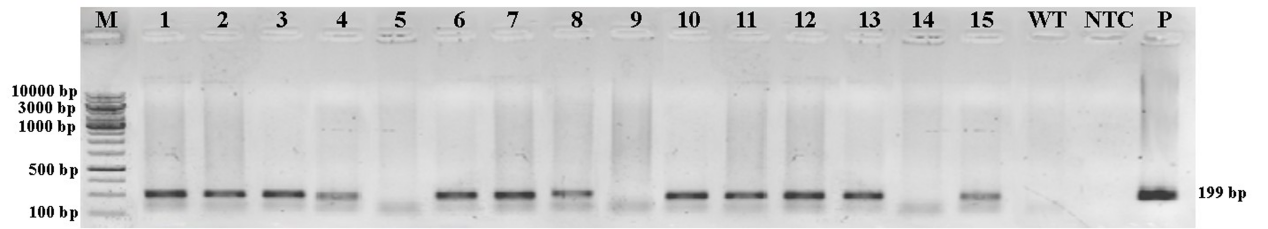


**Supplementary Figure S4.** The original gel image shown in Figure 4B.
